# FGF21 induces autophagy‐mediated cholesterol efflux to inhibit atherogenesis via RACK1 up‐regulation

**DOI:** 10.1111/jcmm.15118

**Published:** 2020-03-30

**Authors:** Lin Xiaolong, Guo Dongmin, Mihua Liu, Wang Zuo, Hu Huijun, Tan Qiufen, Hu XueMei, Lin Wensheng, Pan Yuping, Lin Jun, Zeng Zhaolin

**Affiliations:** ^1^ Department of Pathology Huizhou Third People's Hospital Guangzhou Medical University Huizhou City China; ^2^ Key Laboratory for Arteriosclerology of Hunan Province Institute of Cardiovascular Disease University of South China Hengyang City China; ^3^ Department of infectious Disease Centre for Lipid Research & Key Laboratory of Molecular Biology for infectious Diseases (Ministry of Education) Institute for Viral Hepatitis The Second Affiliated Hospital Chongqing Medical University Chongqing City China; ^4^ Department of Laboratory Medicine First Affiliated Hospital of Gannan Medical University Ganzhou City China; ^5^ Department of Cardiology Nanchuan People’s Hospital Chongqing Medical University Chongqing City China

**Keywords:** atherosclerosis, autophagy, fibroblast growth factor 21, RACK1

## Abstract

Fibroblast growth factor 21 (FGF21) acts as an anti‐atherosclerotic agent. However, the specific mechanisms governing this regulatory activity are unclear. Autophagy is a highly conserved cell stress response which regulates atherosclerosis (AS) by reducing lipid droplet degradation in foam cells. We sought to assess whether FGF21 could inhibit AS by regulating cholesterol metabolism in foam cells via autophagy and to elucidate the underlying molecular mechanisms. In this study, ApoE^−/−^ mice were fed a high‐fat diet (HFD) with or without FGF21 and FGF21 + 3‐Methyladenine (3MA) for 12 weeks. Our results showed that FGF21 inhibited AS in HFD‐fed ApoE^−/−^ mice, which was reversed by 3MA treatment. Moreover, FGF21 increased plaque RACK1 and autophagy‐related protein (LC3 and beclin‐1) expression in ApoE^−/−^ mice, thus preventing AS. However, these proteins were inhibited by LV‐RACK1 shRNA injection. Foam cell development is a crucial determinant of AS, and cholesterol efflux from foam cells represents an important defensive measure of AS. In this study, foam cells were treated with FGF21 for 24 hours after a pre‐treatment with 3MA, ATG5 siRNA or RACK1 siRNA. Our results indicated that FGF21‐induced autophagy promoted cholesterol efflux to reduce cholesterol accumulation in foam cells by up‐regulating RACK1 expression. Interestingly, immunoprecipitation results showed that RACK1 was able to activate AMPK and interact with ATG5. Taken together, our results indicated that FGF21 induces autophagy to promote cholesterol efflux and reduce cholesterol accumulation in foam cells through RACK1‐mediated AMPK activation and ATG5 interaction. These results provided new insights into the molecular mechanisms of FGF21 in the treatment of AS.

## 1 INTRODUCTION

Atherosclerosis (AS) is a chronic and degenerative disease in which lipids accumulate in the walls of large arteries. Macrophages promote foam cell development and plaque formation through the accumulation of cholesterol esters, which is the hallmark of AS.^1^, ^2^ Therefore, enhancing the clearance of cholesterol from macrophage foam cells, especially by increasing the cholesterol efflux from macrophages, is important to reduce plaque lipid build‐up. Cholesterol efflux entails clearing cholesterol from peripheral macrophages, and its transformation into bile in the liver and ultimately into faeces, making it a promising anti‐atherogenic strategy.^3^ Recently, our group found that allicin and curcumin can induce foam cell cholesterol efflux.^4^, ^5^ FGF21 is also reported to be involved in cholesterol efflux.^6^ However, the mechanisms underlying the effects of FGF21 on lipid metabolism in foam cells remain largely unclear.

As a metabolic regulator, FGF21 exhibits outstanding fat‐regulating ability and anti‐AS potential by increasing high‐density lipoprotein (HDL) levels and reducing triglycerides, total cholesterol (TC) and low‐density lipoprotein (LDL) levels in the serum.^7^, ^8^ Recent studies suggested that FGF21 can attenuate the oxidative stress protect against hydrogen peroxide‐induced cytotoxicity by preventing mitogen‐activated protein kinase (MAPK) activation within human umbilical vein endothelial cells (HUVECs).^9^ FGF21 administration reverses oxidative stress induced by a high‐fat diet (HFD) in rats and reduces circulating malondialdehyde, glutathione and superoxide dismutase.^10^ Moreover, some studies demonstrated that FGF21 is anti‐inflammatory. For instance, FGF21 inhibits lipopolysaccharide‐induced NF‐κB activation by promoting I‐κB degradation and impairing p65 translocation to the nucleus.^11^ FGF21 enhances AMPK activation and expression of Sirt1 in alcohol‐treated HepG2 cells^12^ and reduces the expression of C‐reactive protein, tumour necrosis factor α and another inflammatory factor in macrophages.^7^ Emerging evidence indicates that FGF21 reduces AS risk factors and inhibits certain AS‐associated pathogenic mechanisms.^9^, ^10^, ^11^, ^13^ However, the mechanism behind the effect of FGF21 on macrophage foam cells, which are important for AS development, is poorly understood. Our previous study has reported that FGF21 can act on macrophage foam cells to reduce cholesterol accumulation.^6^ Specifically, how FGF21 affects lipid metabolism in macrophage foam cells remain largely unknown. Therefore, an in‐depth exploration of the underlying mechanisms of FGF21 is of great importance for the prevention of AS.

Autophagy is an essential process whereby cells are able to breakdown large portions of the cytoplasm and cellular organelles and recycle them via a catabolic and a highly conserved process.^14^ Lipids are degraded to free cholesterol and fatty acids through autophagy.^15^ Besides, Wang et al^16^ have reported that inhibiting the activation of mTOR activates autophagy and drives cholesterol efflux, thereby decreasing foam cell lipid accumulation, while another study suggested that lysosomal acid lipase mediated autophagy‐mediated regulation of cholesterol efflux.^17^ Furthermore, mTOR inhibitor everolimus selectively depletes macrophages in atherosclerotic plaques by autophagy.^18^ These findings suggest that induction of autophagy is an essential strategy to reduce foam cell lipid accumulation and the prevention of AS.

Activated kinase C receptor 1 (RACK1), a highly conserved intracellular adaptor protein, which was initially discovered to be integrated with Protein kinase C (PKC), is encoded by the *GNB2L1* gene.^19^ As an adaptor protein, RACK1 can provide a platform for the interaction with other proteins and regulate their activities. Studies reported that RACK1 can induce the formation of Atg14L‐Beclin‐1‐Vps34‐Vps15^20^ or interact with ATG5 to promote RACK1‐ATG5 complexes formation,^21^ promote autophagy and inhibit hepatic lipid accumulation. In addition, RACK1 also induces the activation of AMPK to protect the liver from ischaemia‐reperfusion (I/R) injury.^22^ Therefore, we suggested that FGF21 may induce autophagy via RACK1 to enhance lipid degradation, increase cholesterol efflux and reduce lipid concentration within foam cells, thus inhibiting AS.

We observed that FGF21 inhibited AS significantly and that there was involvement of autophagy during this process. Moreover, RACK1 was highly expressed in the atherosclerotic plaque in ApoE^−/−^ mice. Furthermore, the expression of RACK1 was up‐regulated in the foam cells by FGF21. Consequently, autophagy was induced to drive cholesterol efflux and decrease cholesterol accumulation, thus preventing AS. Our findings provide novel information regarding the mechanisms of FGF21 and their role in the prevention and treatment of AS.

## 2 MATERIALS AND METHODS

### 2.1 Reagents

RPMI 1640 medium FBS (C0252) was acquired from Beyotime Biotechnology (Shanghai, China). Phorbol‐12‐Myristate13‐acetate (PMA; P1585) and 3‐methyladenine (3MA, 5 mmol/L; M9281) were purchased from Sigma‐Aldrich. RACK1 siRNA (sc‐36354), ATG5 siRNA (sc‐41445), and primary antibodies such as RACK1 (sc‐17754), beclin‐1 (sc‐11427), LC3 (sc‐398822), ABCA1 (sc‐53482), GAPDH (sc‐47724), β‐actin (sc‐47778) and AMPK (sc‐17754) were acquired from Santa Cruz Biotechnology. Other primary antibodies like anti‐AMPK (2793) and anti–phospho‐AMPK Thr172 (2535) from acquired from Cell Signaling Technology, while p62 primary antibody was supplied by R&D Systems. Acetylated LDL (Ac‐LDL; L8940), FGF21 (ab63277) and secondary goat anti‐mouse IgG‐HRP (ab97023) and goat anti‐rabbit IgG (ab205718) antibodies came from Abcam, while pEGFP‐LC3 was supplied by Cell Biolabs.

### 2.2 Animal model

All animals were killed using carbon dioxide. No anti‐aesthetic agent was used. Ethical guidelines of the Chinese Association for Laboratory Animal Sciences from Directive GB14925‐2010 regarding animal use in scientific studies were followed.

ApoE^−/−^ mice (males, 8 weeks old) were acquired from the Changzhou Card Vince Laboratory Animal Co., Ltd (SPF grade, Certificate No SCXK20150004; Changzhou, China). The Institutional Animal Care and Use Committee at University of South China approved these animal studies. ApoE^−/−^ mice were housed in standard cages with a 12‐hour light/dark cycle in climate‐controlled conditions. The ApoE^−/−^ mice were randomized into 6 groups (n = 10/group). Control, ApoE^−/−^ mice fed with a normal diet; HFD, ApoE^−/−^ mice fed with a HFD for 12 weeks; HFD + NS, ApoE^−/−^ mice fed a HFD with an intraperitoneal (IP) saline for 12 weeks; FGF21 + HFD, ApoE^−/−^ mice fed a HFD with 10 mg/kg/d of FGF21 for 12 weeks; HFD + 3MA, ApoE^−/−^ mice fed a HFD with an IP injection of 3MA (30 mg/kg) for 12 weeks; FGF21 + HFD + 3MA or LV‐RACK neg, ApoE^−/−^ mice fed a HFD with IP injection of 3MA (30 mg/kg) or LV‐RACK shRNA + FGF21 (10 mg/kg/d) for 12 weeks. Animal body weights were recorded weekly, and after 12 weeks of daily injections, animals were killed using carbon dioxide, and blood samples were collected from the ophthalmic venous plexus. The concentrations of serum lipids were determined using instructions provided with the commercial detection kit. The mouse aorta was examined for the presence of atherosclerotic lesions.

### 2.3 Cell culture

THP‐1 cells were grown in RPMI 1640 containing 10% FBS and penicillin/streptomycin in an incubator as described above. Macrophage differentiation was achieved by adding 160 nm/mL PMA to the culture and incubating it for 24 hours. Subsequently, cells were washed with PBS and incubated with a serum‐free medium supplemented using 50 µg/mL ac‐LDL for 48 hours, in order to generate AS‐associated macrophage foam cell model. To elucidate the mechanisms underlying the effects of FGF21, this cell model was treated with ATG5 siRNA, 3MA (5 mmol/L) or RACK1 siRNA prior to FGF21 treatment.

### 2.4 Evaluation of foam cell formation

After removing the supernatants, cells were washed thrice using PBS followed by a 10‐minute fixation step using 4% paraformaldehyde. After an additional wash, cells were placed in 60% isopropanol for 4 minutes. Cells were then stained using Oil Red O solution for 15 minutes that had just been filtered. After washing with 60% isopropanol, counterstaining was done for 40 seconds using haematoxylin. Finally, the stained cells were observed under a Leica phase‐contrast microscope (DMI4000B, Wetzlar, Germany).

### 2.5 Assessment of cellular cholesterol efflux

Cholesterol efflux was assessed based on methods reported previously.^6^ Briefly, 0.2 µCi/mL of [^3^H]‐cholesterol was used for cell labelling in a black microtitre plate and incubated at 37°C overnight. After 24 hours of incubation, cells were washed and resuspended using RPMI supplemented with 0.1% bovine serum albumin (BSA) overnight to equilibrate the cholesterol content. We then measured [^3^H]‐cholesterol in both the media and in the cells via liquid scintillation counting, with efflux being measured based on the formula: 100% × [total media counts/(total cell counts + total media counts)].

### 2.6 Plasmid transfection

After seeding cells atop coverslips in a 24‐well plate and incubating it overnight, 2 µg of the pEGFP‐LC3 plasmid, Opti‐Mem media and Lipofectamine 2000 reagent were added based on provided instructions. Before treatment, the foam cells were grown in RPMI with 10% FBS. Coverslips were then transferred onto slides and were observed using laser scanning confocal microscopy (LSM 880 with Airyscan, Carl Zeiss AG; Overcoaching, Germany).

### 2.7 Western blotting

RIPA lysis buffer was used to isolate cellular proteins at 4°C. The concentration of total protein was detected using a BCA kit (Beyotime Biotech). Protein samples were then separated using 10% or 12% SDS‐PAGE gels, followed by transfer to PVDF membranes (Millipore). Next, TBST (Tris‐buffered saline, 0.1% Tween 20) containing 5% non‐fat milk powder was used for blocking for 2 hours, and then, membranes were incubated overnight at 4°C with anti‐GAPDH, anti‐β‐actin, anti‐RACK1, anti‐LC3, anti‐p62, anti‐beclin‐1 (all 1:1000), anti‐ABCA1 (1:1500), anti‐AMPK (1:1500), and anti‐p‐AMPK (1:500) antibodies. Blots were then washed thrice and probed using secondary antibodies (1:2000) for 2 hours at room temperature. Finally, chemiluminescence was used to visualize protein bands (Automatic Chemiluminescence Image Analysis System, Tanon 4600; Shanghai, China).

### 2.8 HPLC assay

HPLC analysis was carried out as described previously.^6^ PerkinElmer TotalChrom software was used for data analysis.

### 2.9 Morphological assessments

The aortae were removed from killed animals and were stained for 30 minutes with Oil Red O. Samples were then differentiated for 15 minutes in 70% alcohol, washed and then observed. In addition, haematoxylin and eosin (H&E) staining was performed using aortic tissue samples that had been fixed using 10% (w/v) neutral formalin for 24 hours or more. Aortic roots were then processed using standard protocols prior to H&E staining. After that, intimal and medial thicknesses were assessed via an image processing system with a 0.1 mm lens and 400× amplification.

The aorta was placed in 10% neutral buffered formalin overnight. The aortic arch was then opened lengthwise through the lesser curvature and pinned flat en face in a wax‐bottomed dissecting pan. The tissue was stained for 15 minutes using 0.5% Sudan IV solution (ProSciTech, China) in acetone and 70% ethanol (1:1). The tissue was then placed in 80% ethanol for 5 minutes and washed gently with water for several minutes. Digital images of the stained samples were acquired and staining was quantified as a percentage of the total tissue area.

### 2.10 Transfection with small interfering RNA

Human ATG5 or RACK1 siRNA (Santa Cruz Biotechnology) was purchased with target sequences as follows: ATG5‐si: 5′‐GTCCATCTAAGGATGCAAT‐3′ and RACK1‐si: 5′‐GGTCCAGGATGAGAGTCAT‐3′. A scrambled targeting sequence was used as a negative control (Santa cruz, CA, USA). Lipofectamine 2000 (Invitrogen) was used to transfect cells (2 × 10^6^/well) as described above. The efficiency of transfection was verified via real‐time PCR and Western blotting (Figure S1A,B).

### 2.11 Real‐time PCR

Triazolo reagent (Invitrogen) was used to extract total RNA based on provided instructions. Approximately 1 µg of RNA was used to generate cDNA using the Taqman Reverse Transcription Reagent Kit (Invitrogen). To detect the mRNA levels of *RACK1*, *ATG5* and *GAPDH* (control), real‐time PCRs were conducted in a real‐time quantitative PCR system using an Ultra SYBR Mixture kit (CWBIO, China). The following primer sequences were used: RACK1‐F: 5′‐CCCAGATTATTGCCCAGAGA‐3′; RACK1‐R: 5′‐CAATGGATAGCTCACAGCAG‐3′; ATG5‐F: 5′‐GTGCTTCGAGATGTGTGGTTT‐3′; ATG5‐R: 5′‐CCATCCAGAGTTGCTTGTGAT‐3′; GAPDH‐F: 5′‐TGGCCTTCCGTGTTCCTACC‐3′; and GAPDH‐R: 5′‐CGCCTGCTTCACCACCTTCT‐3′. ^△△^Ct method was used for the relative quantitation of *RACK1* and *ATG5* levels, in which *GAPDH* was used as the internal control.

### 2.12 Transmission electron microscopy (TEM)

Transmission electron microscopy was conducted as described in a previous study.^23^ Briefly, 0.1 mol/L sodium cacodylate‐buffered (pH 7.4) 2.5% glutaraldehyde solution was used to fix cells at 4°C for 2 hours, after which the cells were washed thrice using 0.1 mol/L sodium cacodylate‐buffered (pH 7.4) 7.5% sucrose. Next, 1% OsO4 solution was added and the cells were kept for 1.5 hours. Samples were then dehydrated using an ethanol gradient, and the cells were embedded using EMbed 812 (EMS) and cut into ultrathin sections. Next, 2% uranyl acetate was used to stain the cells for 12 minutes, followed by treatment with Reynolds solution (pH 12.4) for 8 minutes. Finally, the stained ultrathin sections were assessed using a FEI Tacna microscope at 120 kV.

### 2.13 Plasma lipid profile evaluation

We used 3% isoflurane to anaesthetize the mice, and the plasma samples were collected from the retro‐orbital plexus. Afterwards, the levels of blood lipids were measured enzymatically (Boster Biological Technology Co., Ltd.), as described previously.^9^

### 2.14 Construction of lentivirus vector

shRNA (GGATGAGACCAACTATGGA) against *RACK1* was ligated into the lentivirus (GenePharma, Shanghai, China). The lentivirus with the negative sequence (GTCACTCACCCTTCGGTTATT), which did not target any gene, was used as a negative control. LV‐RACK1 shRNA was diluted to a total volume of 300 μL containing 4 × 10^7^ pfu and was injected into the tail vein of 8‐week‐old male ApoE^−/−^ mice (once a week for 4 weeks). Control mice were injected with the same dose of empty vector of lentivirus (the efficiency of LV‐RACK1 shRNA is shown in Figure S1C).

### 2.15 Statistical analysis

GraphPad Prism was used for all analyses. Experimental results were assessed via one‐way ANOVA and Student‐Newman‐Keel's (SNK) post hoc multiple comparison tests. Data are presented as means ± SD *P* < .05 was the significance threshold used.

## 3 RESULTS

### 3.1 FGF21 altered blood cholesterol levels and inhibited AS in ApoE^−/−^ mice

Fibroblast growth factor 21 is known to alter lipid profiles as well as to inhibit AS in a few animal models.^7^, ^24^ To verify the reported data, the effects of FGF21 were examined on the lipid profiles of ApoE^−/−^ mice fed with a HFD—the levels of TC, TG, HDL‐C and LDL‐C were measured in these animals (Table 1). Relative to controls, serum TG, TC and LDL‐C remarkably increased, whereas HDL‐C levels markedly decreased (*P* < .05) in the mice with atherosclerosis (HFD group) (Table 1). In addition, body weight of mice in HFD group significantly increased relative to the control group. These results indicated that the mice with atherosclerotic lesions are often accompanied by disturbances in lipid metabolism.

**Table 1:** Effect of Body weight and blood lipid levels on FGF21 in apoE^−/−^ mice. Plasma lipid levels were evaluated enzymatically

	Control	HFD	HFD + NS	HFD + FGF21
Body (g)	20.36 ± 1.98	32.42 ± 1.88^a^	31.78 ± 1.92	22.12 ± 1.95^b^
TG (mmol/L)	2.87 ± 0.08	3.96 ± 0.38^a^	3.92 ± 0.34	2.97 ± 0.05^b^
TC (mmol/L)	4.84 ± 0.16	17.14 ± 0.17^a^	17.09 ± 0.12	9.78 ± 0.15^b^
LDL‐C (mmol/L)	2.72 ± 0.12	8.12 ± 0.15^a^	8.07 ± 0.17	4.09 ± 0.11^b^
HDL‐C (mmol/L)	1.77 ± 0.07	1.09 ± 0.10^a^	1.11 ± 0.11	1.68 ± 0.08^b^

Note

The results are expressed as the mean ± SD in each group.

Abbreviations: HFD: high‐fat diet; NS: normal saline.

^a^ *P* < 0.05 vs control group.

^b^ *P* < 0.05 vs HFD group.

**Figure 1:**
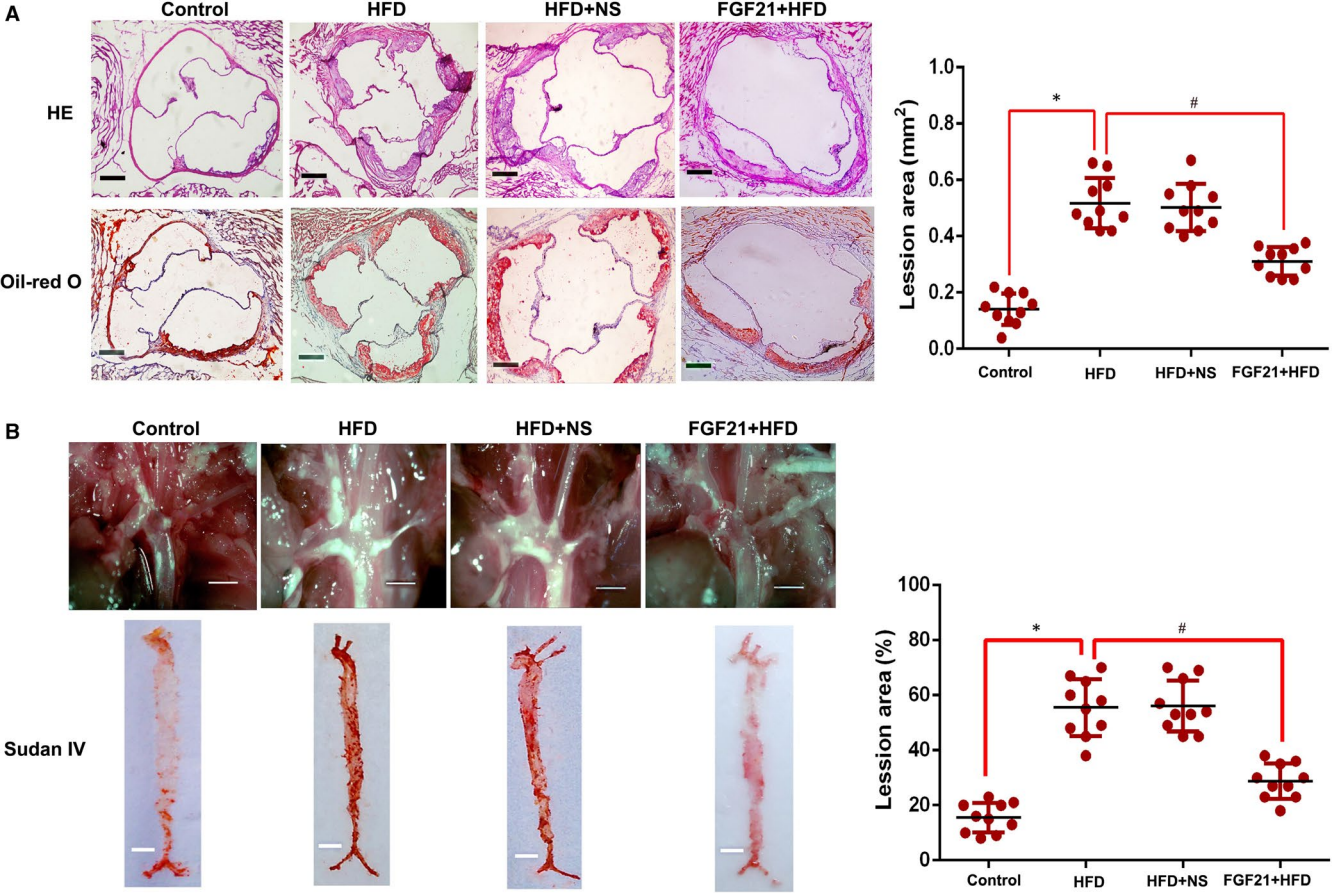
Effects of FGF21 on aortic sinus lesions in the ApoE^−/−^ mice. A, Atherosclerotic plaques in the proximal aorta of ApoE^−/−^ mice stained with Oil Red O and H & E. Original magnification, 40×. Data are presented as mean + SEM (n = 10 per group, SNK post hoc multiple comparison tests); *P < .05 vs control; #*P* < .05 vs AS3; scale bar, 50 μm. B, Lesion area was quantified following whole aortic Sudan IV staining, and a representative image is shown. Original magnification, 5×. Data are presented as mean + SEM (n = 10 per group, SNK post hoc multiple comparison tests); *P < .05 vs control; #*P* < .05 vs FGF21 + HFD; scale bar, 10 mm; HFD, high‐fat diet; NS, physiological saline

Total cholesterol, LDL‐C and TG levels markedly reduced in the FGF21 + HFD group, while HDL‐C levels markedly increased (*P* < .05), relative to HFD group (Table 1). These findings suggested that FGF21 treatment improves lipid metabolism in the ApoE^−/−^ mice fed with a HFD. Moreover, the atherosclerotic lesions in mice with HFD were found to be significantly ameliorated by FGF21 treatment, as observed using aortic H & E and Oil Red O staining (Figure 1A,B). Taken together, FGF21 can alter lipid profiles and inhibit AS development in ApoE^−/−^ mice.

### 3.2 Autophagy is involved in FGF21‐mediated inhibition of AS

**Figure 2:**
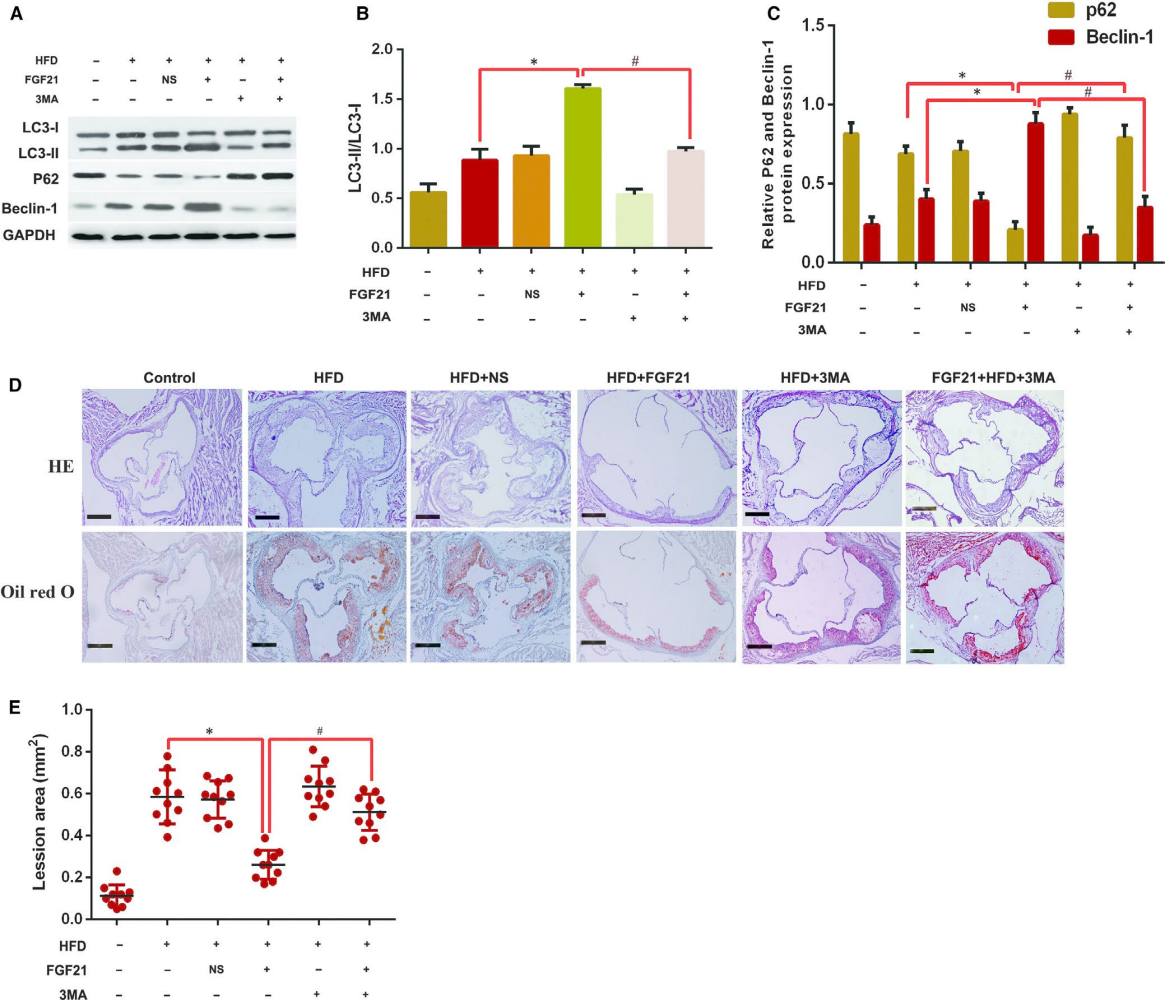
Autophagy is involved in the inhibition of AS by FGF21. ApoE^−/−^ mice were fed a HFD with or without FGF21 and FGF21 + 3MA for 12 wk. A‐C, LC3, beclin‐1 and p62 protein levels were detected using Western blotting. Data are presented as mean ± SD, of 3 experiments. D, E, Oil Red O and H & E staining of an aortic lesion, with representative images shown. Scale bar, 50 μm. Data are presented as mean ± SD (n = 10 per group, SNK post hoc multiple comparison tests), *P < .05 vs HFD group; #*P* < .05 vs FGF21 + HFD + 3MA

Previously, some studies have identified a role of autophagy in AS progression, and FGF21 induces autophagy to protect against myocardial injury in ischaemia‐reperfusion^25^ and diabetic cardiomyopathy.^26^ However, whether autophagy is involved in FGF21‐inhibited AS remains largely unclear. Thus, the levels of autophagy‐related proteins were detected in order to determine whether autophagy can be induced by FGF21 in order to inhibit AS. The results demonstrated that FGF21 treatment increased LC3‐II/LC3‐I ratio and beclin‐1 levels, and lowered p62 protein levels, suggesting that autophagy is induced by FGF21 in ApoE^−/−^ mice (Figure 2A‐C). To further confirm that autophagy is involved in FGF21‐inhibited AS, ApoE^−/−^ mice were administered 3MA (30 mg/kg) prior to FGF21 treatment. We found that beclin‐1, p62 levels, LC3‐II/LC3‐I ratio and the AS region were apparently reversed in the FGF21 + HFD + 3MA group compared with FGF21 + HFD group (Figure 2D‐E). These findings revealed that FGF21 induced autophagy in order to inhibit AS.

### 3.3 FGF21 up‐regulated RACK1 expression to induce autophagy and inhibit AS

**Figure 3:**
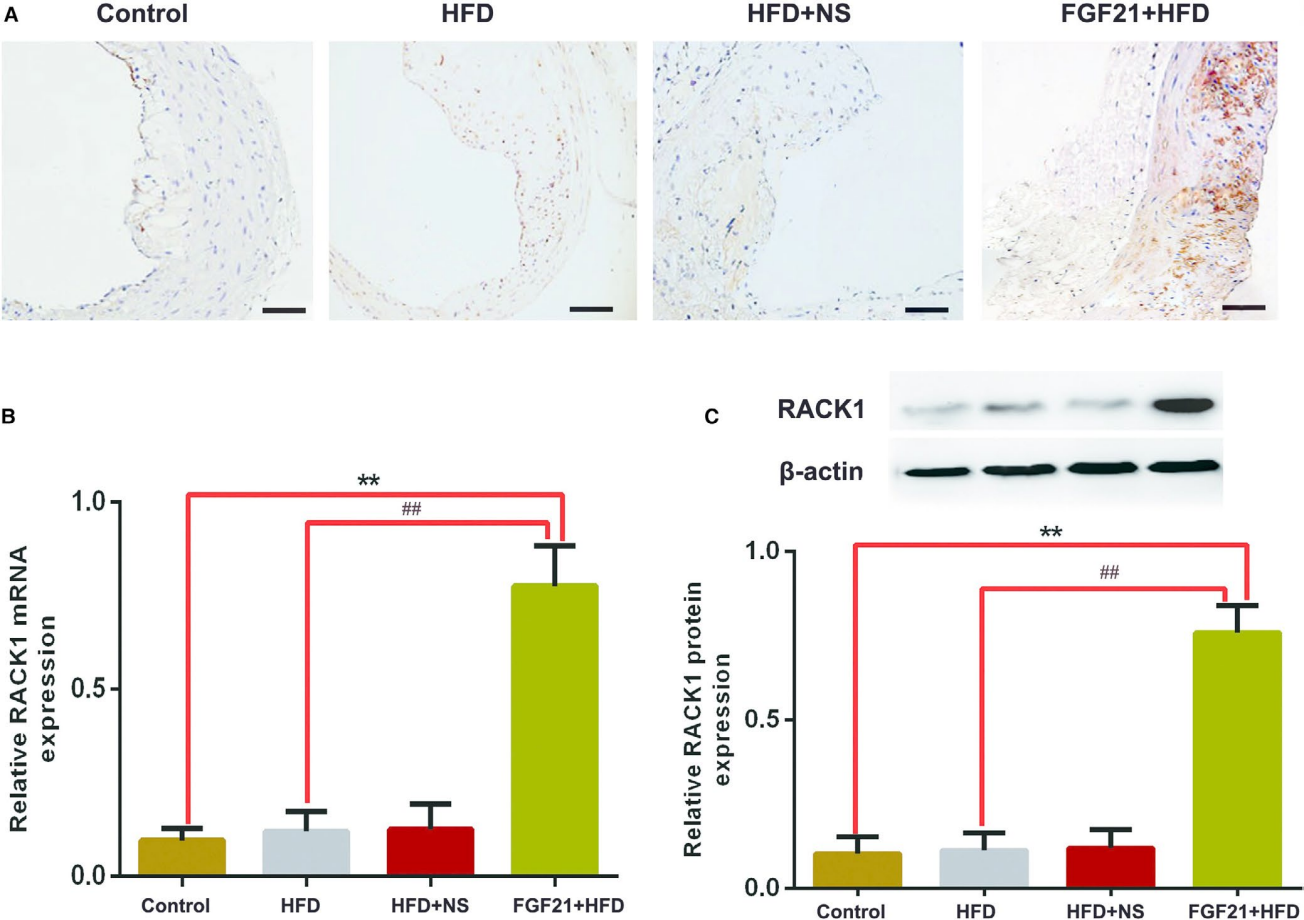
Effects of FGF21 on RACK1 expression in the aortic plaques of apoE^−/−^ mice. A, The expression of RACK1 protein was detected by immunohistochemistry staining. Scale bar, 50 μm. B, The mRNA expression levels of RACK1 were measured by real‐time PCR. C, The protein expression levels of RACK1 were measured using Western blotting. Results are presented as mean ± SD of 3 experiments. **P < .01 vs control group; #*P* < .05, vs FGF21 + HFD group; HFD, high‐fat diet; NS, physiological saline

As a scaffold/adaptor protein, RACK1 can interact with ATG5 to induce autophagy.^21^ RACK1 can induce the formation of Atg14L‐Beclin‐1‐Vps34‐Vps15 and RACK1‐ATG5 complexes, promote autophagy and inhibit hepatic lipid accumulation.^20^ In addition, RACK1 also induces the activation of AMPK to protect the liver from ischaemia‐reperfusion (I/R) injury.^22^ Thus, we propose that FGF21 may increase the expression levels of RACK1 during AS treatment. To test this hypothesis, immunohistochemistry, Western blotting and RT‐PCR analyses were used to detect the mRNA and protein levels of RACK1 in the aortic plaques. RACK1 expression was significantly up‐regulated by FGF21 treatment, suggesting that FGF21‐mediated inhibition of AS progression is dependent on RACK1 expression (Figure 3).

**Figure 4:**
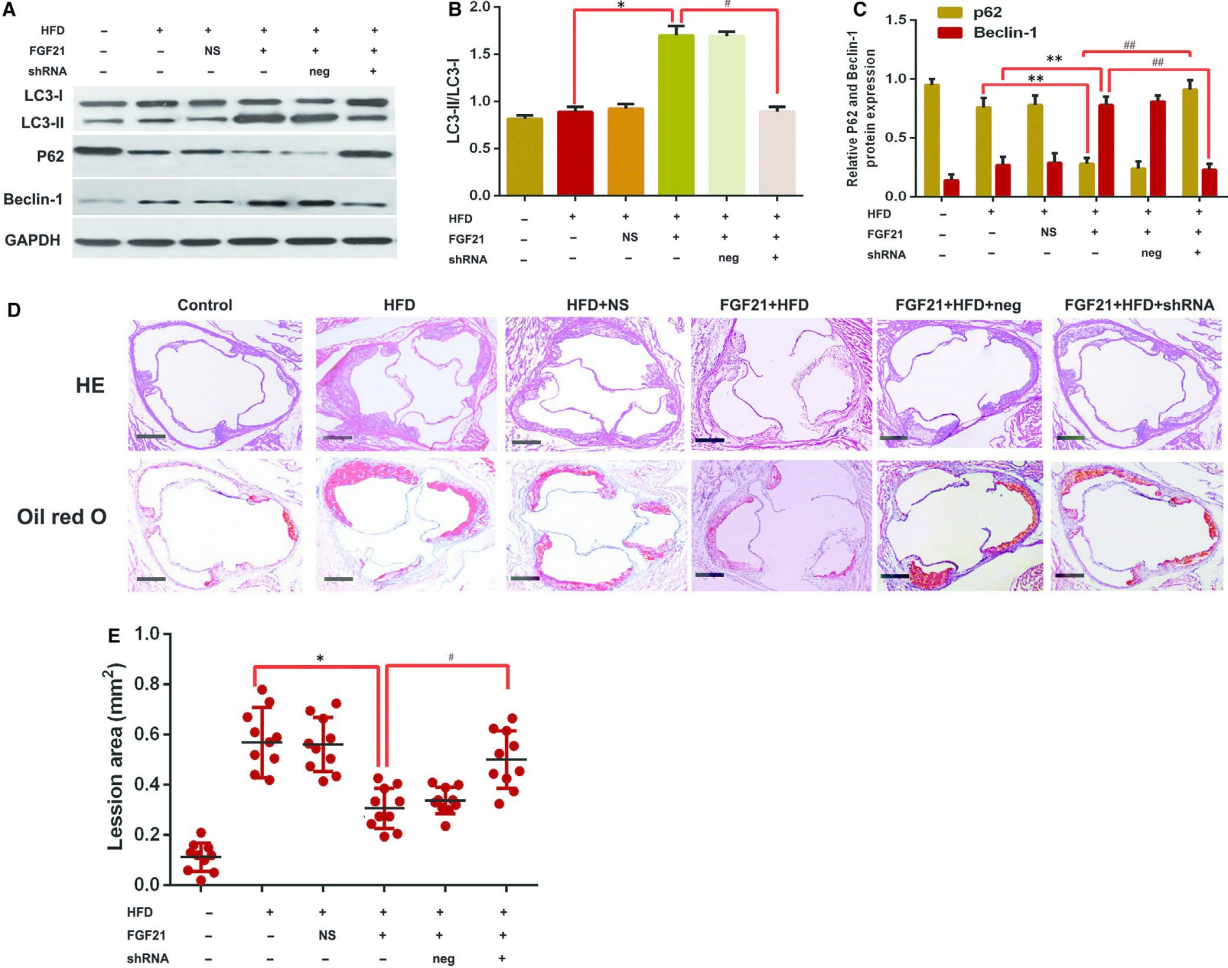
The role of RACK1 in FGF21‐mediated autophagy. ApoE^−/−^ mice were fed a HFD with FGF21 or FGF21 + Ad‐RACK1 shRNA for 12 wk. A‐C, The protein expression levels of LC3, beclin‐1 and p62 protein were detected using Western blotting. Data are presented as mean ± SD of 3 experiments (one‐way ANOVA). **P < .01 vs AS3. D, E, Representative images of H & E and Oil Red O staining of an aortic lesion. Scale bar, 50 μm. Data are expressed as mean ± SD (n = 10 per group, SNK post hoc multiple comparison tests); *P < .05 vs HFD group; #*P* < .05 vs AS3 + LV‐RACK1 shRNA

To further determine whether RACK1‐mediated FGF21 can affect autophagy‐inhibited AS, Ad‐RACK1 shRNA was transfected into ApoE^−/−^ mice for the purpose of RACK1 silencing before FGF21 treatment. The results showed that the effects of FGF21 on LC3‐I to LC3‐II conversion, beclin‐1, p62 expression (Figure 4A‐C) and AS (Figure 4D,E) were reversed. Therefore, these findings suggested that FGF21 up‐regulated RACK1 expression to promote autophagy‐inhibited AS.

### 3.4 FGF21 decreased foam cell lipid accumulation

**Figure 5:**
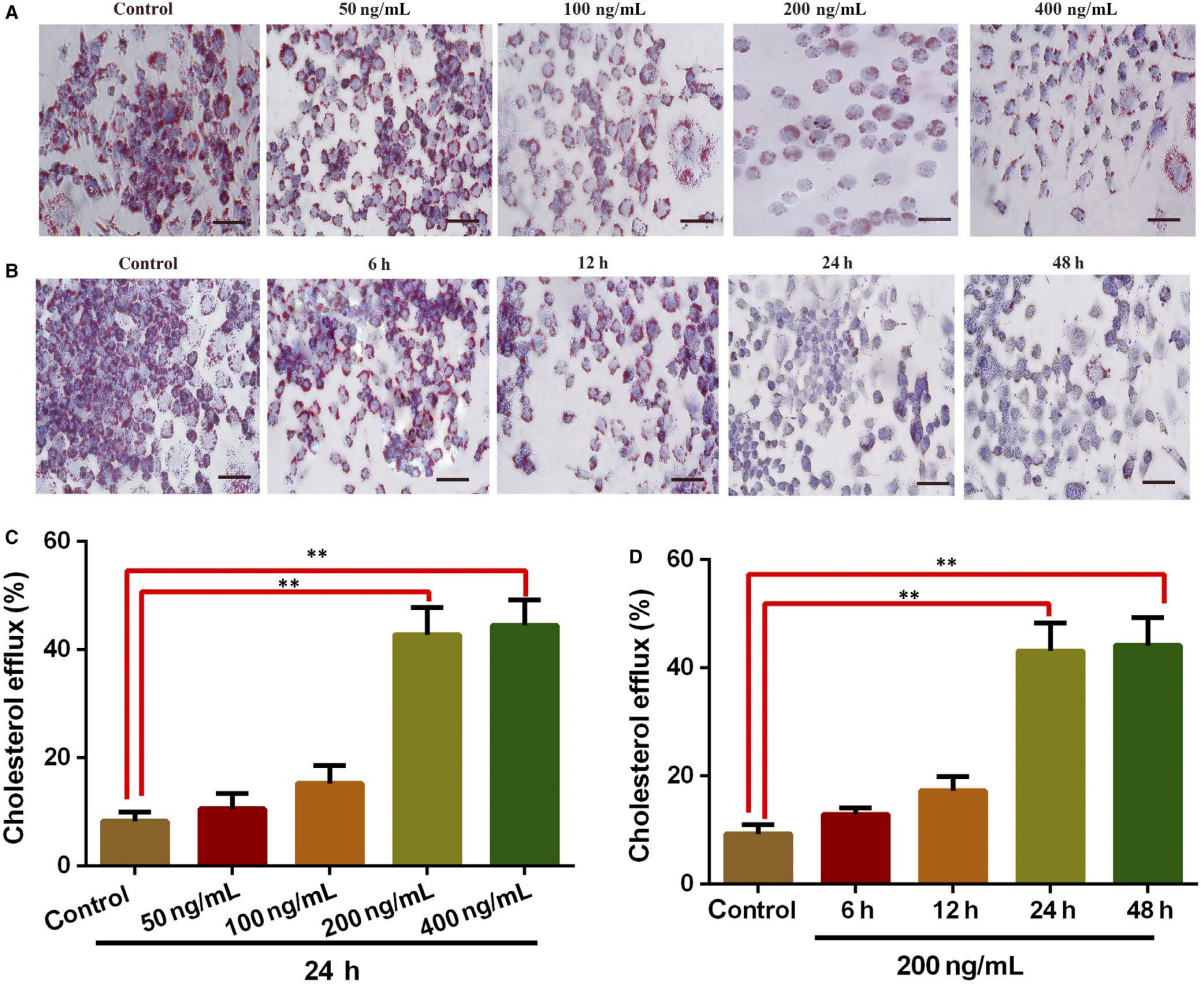
Fibroblast growth factor 21 affects cholesterol accumulation and efflux in foam cells. A, Effects of different concentrations of FGF21 on cholesterol accumulation. Foam cells were treated for 24 h with FGF21 (50, 100, 200 and 400 ng/mL) after which oil Red O staining was used to determine cholesterol accumulation. B, Effects of FGF21 on cholesterol accumulation at different time points. The foam cells were treated with 200 ng/mL of FGF21 (for 0, 6, 12, 24 and 48 h), followed by oil Red O staining and light microscopic examination (400×). Scale bar, 15 μm. Measurements were performed at least three times with similar results. C, Effects of different concentrations of FGF21 on cholesterol efflux. The foam cells were incubated with FGF21 (50‐400 ng/mL) for 24 h. Cholesterol efflux assay was then conducted. D, Effects of FGF21 on cholesterol efflux at different time‐points. The foam cells were treated using 200 ng/mL FGF21 after 0, 6, 12, 24 and 48 h. Cholesterol efflux assay was conducted as described above. Data represent the mean (±SD) of 3 experiments (one‐way ANOVA). **P < .01 vs control

The formation of foam cells is a major hallmark of early‐stage AS and thus can be an appropriate target for therapy.^27^ FGF21 is a metabolic modulator that regulates lipid and glucose metabolism in liver^28^ and adipocytes,^29^ and affects lipid uptake by macrophages.^6^ Thus, FGF21 may alter lipid accumulation in the foam cells in order to suppress AS progression. To explore whether FGF21 can affect lipid accumulation, foam cells were treated with 50‐400 ng/mL of FGF21. Cellular lipid analysis was carried out by HPLC and Oil Red O staining as a means of assessing cholesterol accumulation in the foam cells. It was found that FGF21 (200 and 400 ng/mL) significantly reduced cholesterol ester (CE), TC and free cholesterol (FC) levels (Table 2), as well as cholesterol accumulation (Figure 5A) in foam cells compared to the control group. Similar results were obtained in the foam cells administered with 200 ng/mL of FGF21 and incubated for 24 and 48 hours (Table 3; Figure 5B). Taken together, this showed that FGF21 reduced cholesterol levels as well as lipid accumulation in foam cells.

**Table 2:** The effects of FGF21 on cholesterol content with different concentration in foam cells

	TC (mg/g)	FC (mg/g)	CE (mg/g)	CE/TC (%)
Control	472 ± 26	196 ± 20	276 ± 22	58.5
50 (ng/mL)	465 ± 24	187 ± 19	282 ± 19	59.7
100 (ng/mL)	368 ± 17	147 ± 21	221 ± 18	60.1
200 (ng/mL)	269 ± 19*	102 ± 22*	167 ± 20*	62.1
400 (ng/mL)	267 ± 21*	101 ± 18*	166 ± 23*	62.0

Note

THP‐1 macrophage‐derived foam cells were divided into six groups and cultured in medium containing 50 ng/mL, 100 ng/mL, 200 ng/mL and 400 ng/mL FGF21 for 24 h. HPLC was performed to determine the levels of cellular total cholesterol (TC), free cholesterol (FC) and cholesterol ester (CE). Data are expressed as the mean ± SD from three independent experiments, each of which was performed in triplicate.

* *P* < .05 vs control group.control group:BSA group.

**Table 3:** The effects of 200 ng/mL FGF21 on cholesterol content with different time in foam cells

	TC (mg/g)	FC (mg/g)	CE (mg/g)	CE/TC (%)
Control	498 ± 19	197 ± 16	301 ± 20	60.4
6 (h)	471 ± 24	182 ± 17	289 ± 21	61.3
12 (h)	446 ± 17	171 ± 21	275 ± 23	61.6
24 (h)	271 ± 21*	103 ± 18*	168 ± 18*	62.0
48 (h)	269 ± 25*	102 ± 19*	167 ± 22*	62.1

Note

Foam cells were divided into five groups and cultured in medium containing 200 ng/mL FGF21 for indicated time. The levels of cellular total cholesterol (TC), free cholesterol (FC) and cholesterol ester (CE) were determined by HPLC. Data are expressed as the mean ± SD from three independent experiments, each of which was performed in triplicate.

* *P* < .05 vs control group.controlgroup:BSA group.

Cholesterol efflux in foam cells involves the transport of cholesterol from the vessel wall to the plasma and then to the liver prior for excretion, thereby preventing AS.^30^, ^31^ Thus, we propose that FGF21‐reduced lipid accumulation is dependent on increased cholesterol efflux. As shown in Figure 5C,D, cholesterol efflux markedly increased by FGF21 treatment at 200 and 400 ng/mL concentrations and 200 ng/mL concentration with an incubation for 24 and 48 hours. Altogether, these results strongly suggested that FGF21 reduced lipid accumulation in foam cells through cholesterol efflux.

### 3.5 FGF21 induced autophagic flux to promote cholesterol efflux in foam cells

Recent studies have demonstrated that autophagy is critical for regulating lipid metabolism and is able to reduce lipid accumulation by promoting cholesterol efflux in the foam cells.^32^, ^33^ However, whether FGF21 can induce autophagy to promote cholesterol efflux in the foam cells is yet to be investigated. To better determine how FGF21 affected autophagic flux, the foam cells were incubated with bafilomycin A (it is an autophagy inhibitor that prevents maturation of autophagic vacuoles by inhibiting fusion between autophagosomes and lysosomes) and FGF21. Notably, bafilomycin A + FGF21 treatment group showed a significant LC3‐I to LC3‐II conversion as well as reduced expression of p62 compared to the control group and the groups treated with bafilomycin A or FGF21 alone (Figure S2A‐C). In addition, the proportions of GFP‐labelled autophagosomes increased in bafilomycin A, FGF21, and FGF21 + bafilomycin A groups (Figure S2D). Furthermore, similar results were observed using monodansylcadaverine (MDC) staining and TEM analysis (Figure S2E,F). Overall, these findings showed that FGF21 can induce autophagic flux in foam cells.

**Figure 6:**
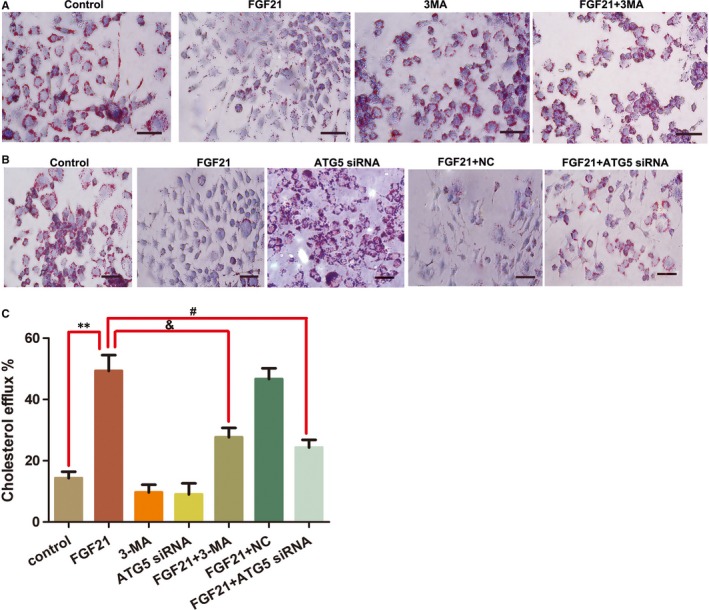
Inhibition of autophagy attenuated FGF21‐induced foam cell cholesterol efflux. A, The foam cells were treated with FGF21 (200 ng/mL) for 24 h with a 3MA (5 mmol/L) pre‐treatment. Oil Red O staining was carried out to determine cholesterol accumulation. Scale bar, 20 μm. The stained cells were examined using light microscopy (400×). B, The foam cells were transfected with scrambled (neg) or ATG5 siRNA and then incubated with 200 ng/mL FGF21 for 24 h. Oil Red O staining was carried out to determine cholesterol accumulation. The stained cells were examined using light microscopy. Scale bar, 30 μm. Measurements were taken at least three times with similar results. C, The foam cells were treated with or without 5 mmol/L of 3MA (for 6 h) pre‐treatment or transfected using scrambled (neg) or ATG5 siRNA, followed by incubation with 200 ng/mL of FGF21 for 24 h. Cholesterol efflux assay was then performed. Results are presented as mean (±SD) of 3 experiments (one‐way ANOVA). **P < .01 vs control; &*P* < .05 vs FGF21 + 3MA; #*P* < .05 vs FGF21 + ATG5 siRNA

Also, to investigate whether autophagy mediates FGF21‐induced cholesterol efflux, the foam cells were treated using FGF21 + 3MA (autophagy inhibitor) or ATG5 siRNA (Table 4 and Figure 6). Pre‐treatment with 3MA significantly inhibited the effects of FGF21 by increasing TC, FC and CE levels, cholesterol accumulation (Table 4; Figure 6A,B) and cholesterol efflux (Figure 6C) in the foam cells. Transient ATG5 knockdown yielded similar results. Taken together, FGF21 promoted cholesterol efflux and reduced lipid accumulation in foam cells via autophagy.

**Table 4:** The role of autophagy in the effects of FGF21 reduced cholesterol content in foam cells

	TC (mg/g)	FC (mg/g)	CE (mg/g)	CE/TC (%)
Control	488 ± 24	193 ± 22	295 ± 19	60.4
FGF21	286 ± 23*, ^#^, ^&^	108 ± 18*, ^#^, ^&^	178 ± 21*, ^#^, ^&^	62.2
3MA	486 ± 24	192 ± 22	294 ± 23	60.4
FGF21 + 3MA	448 ± 21	178 ± 16	270 ± 22	60.2
ATG5 siRNA	458 ± 21	183 ± 17	275 ± 16	60.0
FGF21 + ATG5 neg	288 ± 19	111 ± 21	177 ± 17	61.4
FGF21 + ATG5siRNA	443 ± 21	176 ± 18	277 ± 25	60.3

Note

The foam cells were transfected with scrambled (neg) or ATG5 siRNA and then incubated with 200 ng/mL FGF21 for 24 h. HPLC assay was subsequently performed. Data are expressed as the mean ± SD from three independent experiments, each of which was performed in triplicate.

* *P* < .05 vs control group.

^#^ *P* < .05 vs FGF21 + 3MA group.

^&^ *P* < .05 vs FGF21 + ATG5siRNA group.

### 3.6 FGF21 induced autophagy to promote cholesterol efflux in foam cells via RACK1 up‐regulation

**Figure 7:**
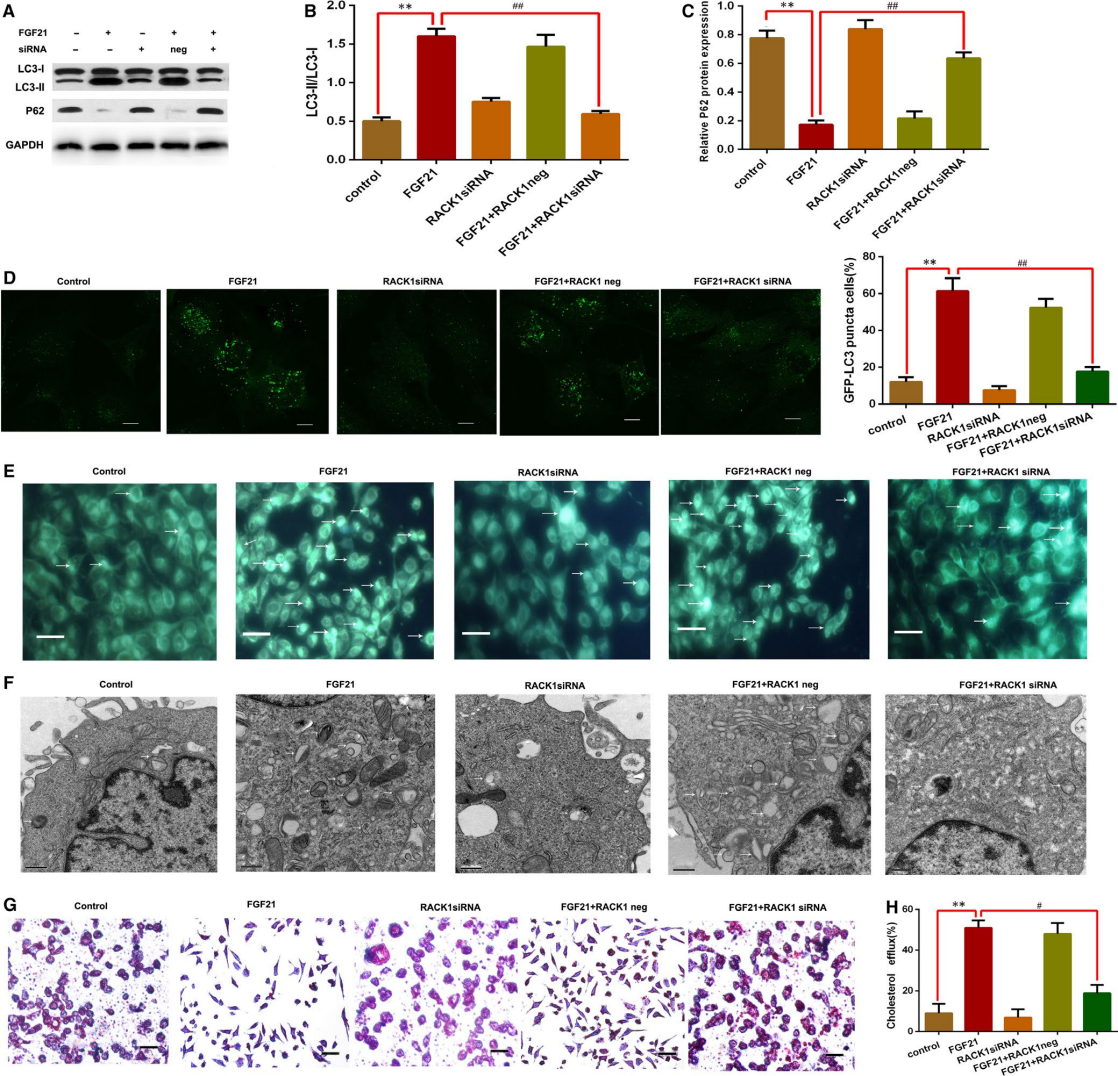
Fibroblast growth factor 21‐induced autophagy increased foam cell cholesterol efflux via RACK1 expression. A‐C, The foam cells were transfected using scrambled (neg) or RACK1 siRNA for 12 h and then incubated with 200 ng/mL of FGF21 for 24 h. LC3 and p62 were measured using Western blotting. D, Punctuated GFP‐LC3 protein was examined using laser scanning microscopy. Scale bar, 5 μm. E, Autophagosomes were assessed using MDC staining and fluorescence microscopy, with representative cell images at the indicated time‐points. Scale bar, 10 μm; white arrow, autophagosome. F, Autophagosomes were assessed using TEM, with images of representative cells shown from indicated time points. Scale bar, 0.5 μm; white arrow, autophagosome. G, Cholesterol accumulation was determined using Oil Red O staining, followed by light microscopic examination. Scale bar, 15 μm; original magnification, 400×. Measurements were conducted three or more times and gave similar results. H, Effect of RACK1 siRNA on FGF21‐induced cholesterol efflux in foam cells. All results are presented as means (±SD) of 3 experiments (one‐way ANOVA). **P < .01 vs controls; #*P* < .05 vs FGF21 + RACK1 siRNA

Based on the above experiments, RACK1 has been identified to promote autophagy and is involved in the inhibition of AS by FGF21, but the underlying mechanisms remain unclear. Recent evidence suggests that RACK1 promotes autophagy‐reduced lipid accumulation by enhancing autophagy‐initiation complex formation in the liver.^20^ Hence, we speculated that FGF21 up‐regulates RACK1 to induce autophagy, increase cholesterol efflux and decrease lipid accumulation in the foam cells. To prove this conjecture, we first detected RACK1 expression levels in the foam cells with FGF21 treatment, showing that FGF21 increased RACK1 expression levels (Figure S1D). Next, the foam cells were pre‐treated with RACK1 siRNA. Our results demonstrated that RACK1 knockdown significantly suppressed FGF21‐induced autophagy, decreased LC3‐I to LC3‐II conversion, up‐regulated p62 expression (Figure 7A‐C) and inhibited autophagosome formation (Figure 7D‐F). Furthermore, FGF21 decreased the levels of TC, FC and CE, reduced cholesterol accumulation and increased cholesterol efflux in foam cells, but these effect also reversed by RACK1 knockdown (Table 5; Figure 7G,H). These data indicated that FGF21 induced autophagy to promote cholesterol efflux in foam cells via RACK1 up‐regulation.

**Table 5:** The role of RACK1 in the effects of FGF21 on cholesterol content in foam cells

	TC (mg/g)	FC (mg/g)	CE (mg/g)	CE/TC (%)
Control	490 ± 21	195 ± 17	295 ± 14	60.2
FGF21	279 ± 15*, ^#^	105 ± 19*, ^#^	174 ± 22*, ^#^	62.3
RACK1 siRNA	481 ± 24	188 ± 22	293 ± 19	60.9
FGF21 + RACK1neg	284 ± 19*, ^#^	108 ± 19*, ^#^	176 ± 22*, ^#^	62.0
FGF21 + RACK1siRNA	440 ± 24	178 ± 21	262 ± 17	60.5

Note

The foam cells were transfected using scrambled (neg) or RACK1 siRNA for 12 h and then incubated with 200 ng/mL of FGF21 for 24 h, HPLC assay was subsequently performed. Data are expressed as the mean ± SD from three independent experiments, each of which was performed in triplicate.

* *P* < .05 vs con group.

^#^ *P* < .05 vs FGF21 + RACK15 siRNA group.

### 3.7 Roles of RACK1 in regulating AMPK activation, ABCA1 expression and RACK1‐ATG5 interaction

AMPK is important in autophagy^34^ and regulating ABCA1 expression.^35^ Moreover, RACK1 interacted with ATG5‐mediated autophagy. More recently, it was shown that AMPK pathway is activated by RACK1 to protect against hepatic I/R injury.^22^ Therefore, we suggested that RACK1 may activate AMPK and interact with ATG5 to regulate FGF21‐induced autophagy and promote foam cell cholesterol efflux. In the present study, RACK1 siRNA was used in foam cells, showing that knocking down RACK1 greatly reduced FGF21‐induced AMPK activation and ABCA1 expression (Figure S3A‐C). Additionally, RACK1 knockdown disrupted the interaction between RACK1 and ATG5 (Figure S3D,E). These data suggested that FGF21‐induced autophagy promoted cholesterol efflux via RACK1 up‐regulation, in order to activate AMPK and interact with ATG5 in the foam cells. Furthermore, the activation of AMPK also up‐regulated the expression levels of ABCA1.

## 4 DISCUSSION

Fibroblast growth factor 21 is a hormone‐like growth factor that lowers the levels of glucose and lipids in non‐human primate and rodent models. Administration of FGF21 has greatly improved the lipo‐profiles, by decreasing LDL‐C and elevating HDL‐C in rodent models.^36^ Recent in vitro and in vivo works suggest that FGF21 can inhibit AS by attenuating inflammation and inhibiting oxidative stress.^36^, ^37^ However, how FGF21 affects lipid metabolism in foam cells is still elusive. Our findings suggested that FGF21 may serve as an essential factor for inhibiting AS progression. More importantly, we found that induction of autophagy can be considered as a novel mechanism of FGF21 to reduce cholesterol accumulation in foam cells and prevent subsequent AS development.

Numerous studies have demonstrated that FGF21 is effective in ameliorating the metabolic syndrome, lowering LDL‐C and improving other related diseases and conditions such as type 2 diabetes, non‐alcoholic steatohepatitis, obesity and chronic inflammation.^36^, ^38^, ^39^ We have found FGF21 to be able to cause a marked improvement in the lipo‐profiles (reducing LDL‐C and elevating HDL‐C) and prevent AS in ApoE^−/−^ mice. In this respect, the increased circulating FGF21 levels may serve as the body's defence mechanism to prevent vascular damage in patients and rodents with AS. Up‐regulation of FGF21 seems a compensatory mechanism to protect against hyperglycaemia‐induced cardiomyopathy and acetaminophen‐induced acute liver injury. To support this contention, we found that oral FGF21 administration for 12 weeks significantly reduced the atherosclerotic lesions in ApoE^−/−^ mice, suggesting that FGF21 exhibited potent hepatoprotective and anti‐hyperlipidemic properties. FGF21 markedly decreased cholesterol levels in the foam cells, based on Oil Red O staining and cholesterol quantification. The results of cholesterol efflux assay showed that FGF21 prevented foam cell formation through induction of cholesterol efflux. Taken together, these data confirmed that FGF21 is a promising candidate for the treatment of AS by regulating lipid metabolism in macrophage foam cells.

Autophagy is an important conserved recycling process necessary to maintain energy balance in the cells, serving a critical function of lipid regulation in obesity and AS.^15^, ^17^, ^40^ However, the role of autophagy in the inhibition of AS by FGF21 is not well understood. Herein, our data revealed that FGF21 increased LC3‐II/LC3‐I ratio, up‐regulated the expression of beclin‐1, down‐regulated p62 protein levels and inhibited AS development in HDF‐fed ApoE^−/−^ mice. In addition, these effects were successfully reversed by the autophagy inhibitor 3‐MA, indicating that autophagy may be involved in the inhibition of AS by FGF21. Macrophage foam cell formation is a central hallmark of AS, and autophagy is a potential target for eliminating foam cells. Yao et al^41^ suggested that activation of autophagy can reduce lipid concentration in foam cells. Therefore, autophagy may be involved in FGF21‐induced cholesterol efflux to reduce cholesterol accumulation in the foam cells. In this work, FGF21 drove autophagy and cholesterol efflux, and decreased intracellular content of cholesterol, triglycerides and lipid droplets of the foam cells. However, these effects of FGF21 on cholesterol efflux and lipid accumulation were partially inhibited by 3MA and ATG5 siRNA pre‐treatment, which was consistent with previous findings that defective autophagy increased apelin 13‐induced lipid storage in foam cells.^40^ Overall, these results suggested that FGF21 partly induced autophagy to increase cholesterol efflux and reduce foam cell cholesterol accumulation, thus inhibiting AS progression.

Recently, several studies have reported that RACK1 interacts with ATG5 and contributes to autophagy.^20^, ^21^, ^42^ Additionally, RACK1 enhances the formation of Atg14L‐Beclin 1‐Vps34‐Vps15 complex, promotes autophagy‐regulated lipid metabolism and prevents increased lipid storage in the liver.^20^ The interaction of lipid droplets and autophagosomes can play a key role in macrophage lipid metabolism. Accumulation of CEs caused by deficiency of autophagy may result in foam cell formation, which occurs early in AS.^30^ Our data found that RACK1 expression was up‐regulated in ApoE^−/−^ mice treated with FGF21, and RACK1 silencing reversed the effects of FGF21 on autophagy and AS. In addition, RACK1 prevented the increase in lipid storage by mediating AMPK activation and ATG5 interaction to induce autophagy in macrophage‐derived foam cells (Figure S3). The involvement of AMPK in autophagy has been previously identified.^6^, ^43^ Here, we have showed that AMPK is a substrate of RACK1, which is phosphorylated at T172. These results showed that RACK1 is involved in the inhibition of AS by FGF21 through AMPK activation and ATG5 interaction to promote autophagy‐induced cholesterol efflux and reduce foam cell cholesterol accumulation.

Improving cholesterol efflux from foam cells represents an efficient strategy for reducing cholesterol accumulation in AS.^44^ In this study, the results of HPLC assay and Oil Red O staining showed that FGF21 significantly decreased cholesterol levels, reduced cholesterol accumulation and suppressed foam cell development. ABCA1 regulated cellular cholesterol efflux to apolipoprotein A‐I (apoA‐I) (free lipid) and production of HDL, thus inhibiting foam cell formation and AS development. We found that FGF21 increased ABCA1 expression, which was also suppressed by RACK1 siRNA treatment. As mentioned earlier, RACK1 phosphorylated AMPK, and our previous work implied that AMPK activation induced foam cell ABCA1 expression. This indicated that RACK1 mediated both AMPK activation and ABCA1 up‐regulation, which may be attributed to the role of FGF21 in autophagy induction, cholesterol efflux and lipid metabolism. Above all, FGF21 induced autophagy and ABCA1 expression to promote cholesterol efflux, reduce cholesterol accumulation and prevent subsequent AS via RACK1 up‐regulation.

In summary, this study established a correlation between autophagy and lipid metabolism in macrophage foam cells and AS development. Our results suggested that FGF21 could promote cholesterol efflux to reduce cholesterol accumulation in foam cells, thus inhibiting AS progression (Figure S4). The mechanism is as follows—on the one hand, RACK1 mediated FGF21‐induced autophagic activity in macrophage foam cells via ATG5 interaction and, on the other hand, FGF21 increased RACK1 expression to induce AMPK activation and then promote ABCA1 expression (Figure S4). Collectively, our results offered novel insights into the roles and mechanisms of FGF21 in the development of AS and suggested that RACK1 may be a therapeutic target for AS treatment.

### 4.1 Inadequacies of the article

In the current study, we only explore the role of FGF21 in inhibiting AS induced by autophagy, but the mechanism of the FGF21‐regulated autophagy remains to be completely elucidated. The following questions are still unanswered—1. How FGF21 increasing RACK1 expression and what is the underlying mechanism? 2. How is RACK1 involved in the formation of the autophagic vacuole or the autolysosome? 3. What is the mechanism underlying the regulation of LC3 and P62 expression by RACK1? These caveats need to be further addressed. FGF21 improved the lipid profiles and increased the adiponectin levels in diabetic and obese subjects who were predisposed to cardiovascular disease (CVDs). We identified a potential role for FGF21 analogs in the treatment of atherosclerosis. However, the administered doses were up to 300 times the normal plasma levels of FGF21. The requirement for supraphysiological doses of FGF21 to halt the pathogenesis of these diseases is consistent with FGF21 resistance in type 2 diabetes and CVDs. Further studies are needed to determine whether FGF21 resistance is involved in the pathogenesis of diabetes and atherosclerosis, and if there is a mechanism to overcome this resistance. Recently, clinical studies have indicated that FGF21 levels increase in atherosclerosis. On the other hand, FGF21 therapy is accompanied by lipid‐lowering effects in non‐human primates and reduced atherosclerotic plaque formation in mice. Due to its anti‐oxidative, anti‐inflammatory, lipid‐lowering and adiponectin‐increasing effects, FGF21 directly or indirectly represses signalling pathways that lead to atherosclerosis. However, a number of these studies were conducted in animals. As there is difference in atherosclerosis susceptibility between humans and rodent, further studies are needed to confirm the therapeutic role of FGF21 against atherosclerosis in humans or large humanoid animals such as pigs. Also, further prospective studies are needed to clarify whether FGF21 can be used as a predictive biomarker to identify individuals at high risk of atherosclerosis in atherosclerosis‐associated diseases and whether FGF21 therapy can reduce the risk of atherosclerosis in these diseases.

## CONFLICT OF INTEREST

The authors declare that they have no conflict of interest.

## AUTHOR CONTRIBUTIONS

Zhaolin Zeng and Dongmin Guo performed atherosclerosis experiments. Zuo Wang, Mihua Liu and Huijun Hu designed and conducted animal experiments. Qiufen Tan and XueMei Hu provided assistance with data acquisition, data analysis and statistical analysis. Wensheng Lin and Yuping Pan carried out literature search and data acquisition and edited the manuscript. Jun Lin and Jie Gao carried out the study and collected important background information. Xiaolong Lin designed and supervised the project and edited the manuscript.

## ETHICAL APPROVAL

The guidelines of the local ethical and Chinese Association for Laboratory Animal Sciences from Directive GB14925‐2010 regarding use of animals in scientific studies were followed.

## Supporting information

Figure S1

Figure S2

Figure S3

Figure S4

Supplementary Material

## Data Availability

The data that support the findings of this study are available from the corresponding author upon reasonable request.
