# Supplementary figures and images for: FGF21 induces autophagy‐mediated cholesterol efflux to inhibit atherogenesis via RACK1 up‐regulation
Source: J Cell Mol Med. 2020 Mar 30;24(9):4992–5006. doi: 10.1111/jcmm.15118 (PMC7205825; doi:10.1111/jcmm.15118)

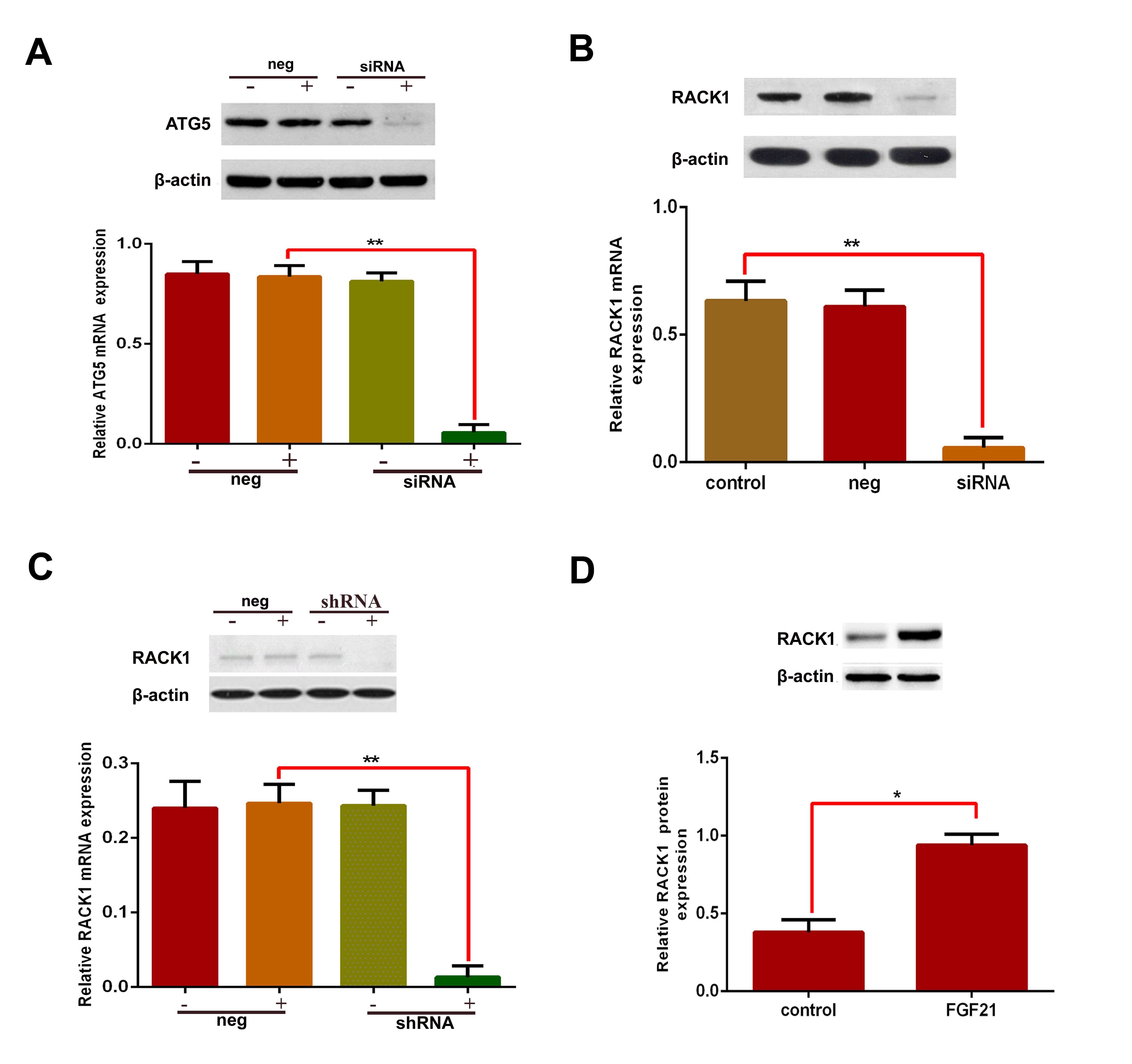

Supplement: Supplementary file 1 — Figure S1 [file JCMM-24-4992-s001.tiff]

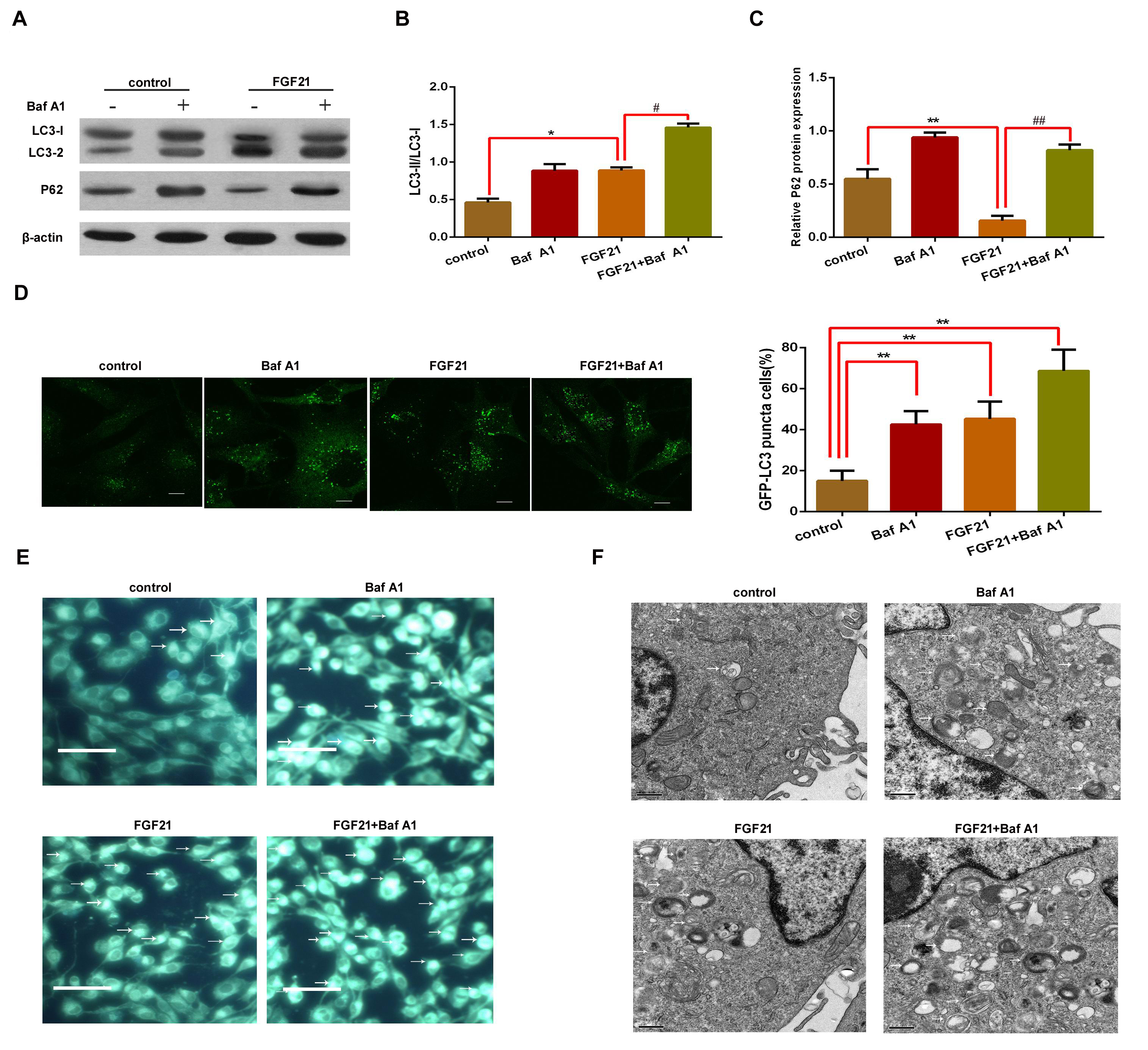

Supplement: Supplementary file 2 — Figure S2 [file JCMM-24-4992-s002.tiff]

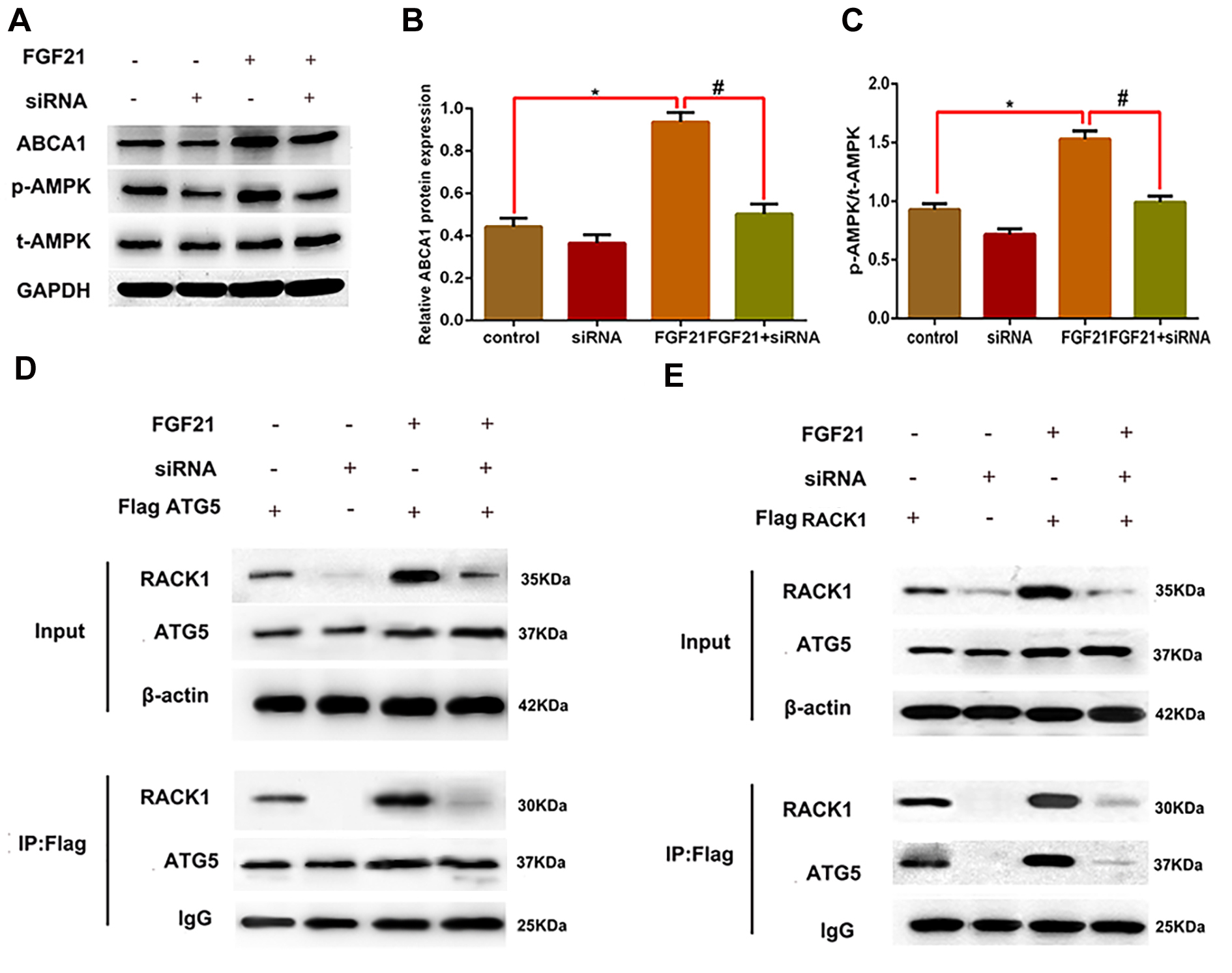

Supplement: Supplementary file 3 — Figure S3 [file JCMM-24-4992-s003.tiff]

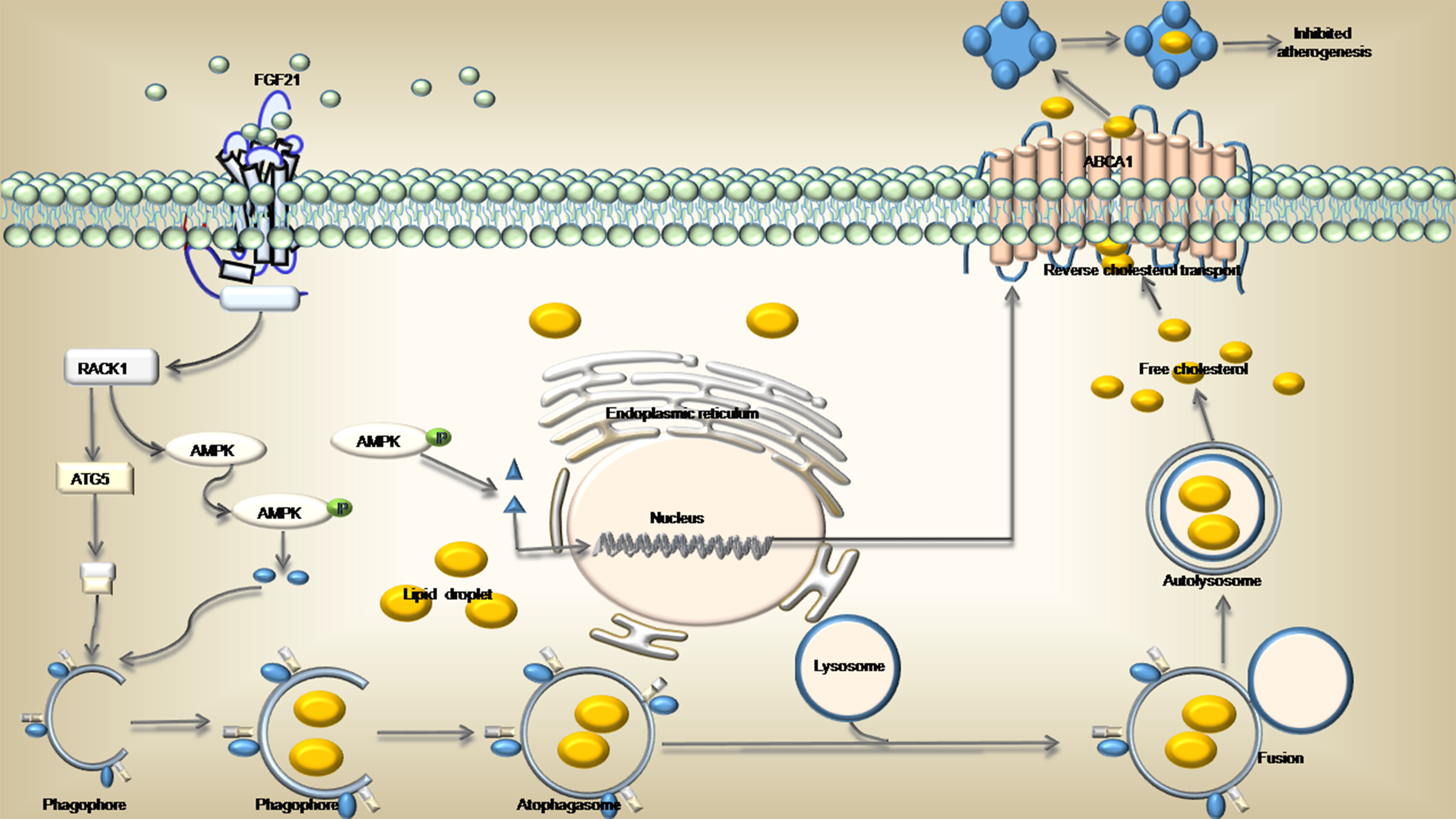

Supplement: Supplementary file 4 — Figure S4 [file JCMM-24-4992-s004.tiff]
