# Supplementary material for: FGF21 induces autophagy‐mediated cholesterol efflux to inhibit atherogenesis via RACK1 up‐regulation
Source: J Cell Mol Med. 2020 Mar 30;24(9):4992–5006. doi: 10.1111/jcmm.15118 (PMC7205825; doi:10.1111/jcmm.15118)
Supplement: Supplementary file 5 — Supplementary Material [file JCMM-24-4992-s005.doc]

**FGF21 induces autophagy-mediated cholesterol efflux to inhibit atherogenesis via RACK1 up-regulation**

Zhaolin Zeng^2,3^,Dongmin Guo^4#^, Mihua Liu^3^,Zuo Wang^4#^, Huijun Hu^1^,Qiufen Tan^1^,XueMei Hu^1^, Wensheng Lin^1^, Yuping Pan^1^,Jun Lin^1^, Xiaolong Lin^1*^

^1^Department of Pathology, Huizhou Third People's Hospital, Guangzhou Medical University, Huizhou City, Guangdong Province 516001, China.

^2^Department of Cardiology, Nanchuan People’s Hospital, Chongqing Medical University, , Chongqing, 408499,China.

^3^ Centre for Lipid Research & Key Laboratory of Molecular Biology for infectious Diseases (Ministry of Education), Institute for Viral Hepatitis, Department of infectious Disease, The Second Affiliated Hospital, Chongqing Medical University, Chongqing City 400016, China.

^4^Key Laboratory for Arteriosclerology of Hunan Province, Institute of Cardiovascular Disease, University of South China, Hengyang City, Hunan Province 421001, China.

^#^Contributed equally to this work

^*^**Corresponding author：**
Dr.Xiao-Long Lin, Department of Pathology, Huizhou Third People's Hospital, Guangzhou Medical University, Huizhou City, Guangdong Province 516001, China. E-mail: [xiaolong880101@126.com](mailto:493814078@qq.com)

**Supplementary Figure Legends**

**Supplement Figure.1.** (A)The efficiency of ATG5 siRNA transfection in foam cells.Cells (2×10^6^cells/well) were transfected ATG5 siRNA or ATG5 scrambled sequence(negative control) for 24h. the expression of ATG5 mRNA and protein were analysis by real time PCR and westernblotting.(B)The efficiency of RACK1 siRNA transfection in foam cells.Cells (2×10^6^cells/well) were transfected RACK1 siRNA or RACK1 scrambled sequence(negative control) for 24h. the expression of RACK1mRNA and protein were analysis by real time PCR and western blotting.Data represent means (±SD) of triplicate experiments(one-way ANOVA).**,*P*<0.01,vs neg group.neg siRNA scrabling.(C)The efficiency of LV-RACK1shRNA transfection in apoE-/- mice vascular. 4×10^7^ pfu/kg body weight of the LV-RACK1 shRNAand empty vector were intravenous injection of apoE-/- mice. the expression of RACK1mRNA and protein were analysis by real time PCR and westernblotting.Data represent means (±SD) of triplicate experiments(one-way ANOVA). **,*P* <0.01,vs neg group.neg:LV-shRNA scrabling.(D)Effect of FGF21 on RACK1 protein expression in foam cells.THP-1macrophage-derived foam cells were incubated with 200ng/ml FGF21 for 24h, the expression of RACK1 and ABCA1 protein were detect by western blot. Data represent means (± SD) of triplicate experiments (one-way ANOVA). *,*P*<0.05,vs control.

**Supplement Figure.2. Effects of FGF21 on autophagy flux in foam cells.** Effects of FGF21 on autophagy flux in foam cells. Foam cells were treated using 20 nM Baf A1 for 4 h prior to FGF21 (200 ng/ml) treatment. (A-C) LC3 and p62 levels were assessed via Western blotting.(D) FGF21-induced GFP-LC3 protein punctate distribution. The cells were transfected for 12 hours using a GFP-LC3 plasmid and followed by treatment FGF21 for 24 h. The cells were then examined by laser scanning microscopy. Scale bar 5 μm. Data represent means (± SD) of triplicate experiments(one-way ANOVA). **,*P* < 0.01 vs. controls; #,*P* < 0.05. vs FGF21+Baf A1. (E) Autophagosomes were assessed via MDC staining and fluorescence microscopy. Scale bar 15μm and 0.5μm, white arrow:autophagosome. (F) Autophagosomes were visualized in more detail using TEM. Scale bar 0.5 μm. white arrow:autophagosome.Representative images are shown for all samples at indicated time points.

**Supplement Figure.3. Roles of RACK1 in regulating AMPK activation, ABCA1 expression, and RACK1-ATG5 interaction.** (A-C) The foam cells were transfected using scrambled (neg) or RACK1 siRNA for 12 h, followed by treatment with 200 ng/ml FGF21 for 24 h. Western blotting was performed to assess p-AMPK, t-AMPK, and ABCA1 levels. Data are presented as mean (± SD) of 3 experiments (one-way ANOVA). *, *P* < 0.05 vs. controls; #, *P* < 0.05 vs. FGF21 + RACK1 siRNA. (D) The foam cells were co-transfected using Flag-ATG5 and/or RACK siRNA for 12 h, followed by incubation with 200 ng/ml FGF21. Following Flag immunoprecipitation, ATG5 and RACK1 immunoblotting was conducted, with β-Actin used as a loading control. (E) The foam cells were co-transfected using Flag-RACK1 and/or RACK siRNA for 12 h, followed by treatment with 200 ng/ml FGF21. Immunoprecipitation and immunoblotting were then performed as described above.

**Supplement Figure. 4. The mechanism used by FGF21 to increase RACK1 expression and cholesterol efflux.** FGF21 increased RACK1 expression, and then induced AMPK activation and its interaction with ATG5 to activite autophagy, contributing to cholesterol ester degradation. Furthermore, AMPK activation also promoted ABCA1 expression. Taken together, FGF21 enhanced cholesterol efflux and reduced foam-cell cholesterol accumulation, thus inhibiting AS progression.
